# An Unexpected Role for the Clock Protein Timeless in Developmental Apoptosis

**DOI:** 10.1371/journal.pone.0017157

**Published:** 2011-02-17

**Authors:** Linda P. O'Reilly, Simon C. Watkins, Thomas E. Smithgall

**Affiliations:** 1 Department of Microbiology and Molecular Genetics, University of Pittsburgh School of Medicine, Pittsburgh, Pennsylvania, United States of America; 2 Department of Cell Biology and Physiology, University of Pittsburgh School of Medicine, Pittsburgh, Pennsylvania, United States of America; Centro Cardiologico Monzino, Italy

## Abstract

**Background:**

Programmed cell death is critical not only in adult tissue homeostasis but for embryogenesis as well. One of the earliest steps in development, formation of the proamniotic cavity, involves coordinated apoptosis of embryonic cells. Recent work from our group demonstrated that c-Src protein-tyrosine kinase activity triggers differentiation of mouse embryonic stem (mES) cells to primitive ectoderm-like cells. In this report, we identified Timeless (Tim), the mammalian ortholog of a *Drosophila* circadian rhythm protein, as a binding partner and substrate for c-Src and probed its role in the differentiation of mES cells.

**Methodology/Principal Findings:**

To determine whether Tim is involved in ES cell differentiation, Tim protein levels were stably suppressed using shRNA. Tim-defective ES cell lines were then tested for embryoid body (EB) formation, which models early mammalian development. Remarkably, confocal microscopy revealed that EBs formed from the Tim-knockdown ES cells failed to cavitate. Cells retained within the centers of the failed cavities strongly expressed the pluripotency marker Oct4, suggesting that further development is arrested without Tim. Immunoblots revealed reduced basal Caspase activity in the Tim-defective EBs compared to wild-type controls. Furthermore, EBs formed from Tim-knockdown cells demonstrated resistance to staurosporine-induced apoptosis, consistent with a link between Tim and programmed cell death during cavitation.

**Conclusions/Significance:**

Our data demonstrate a novel function for the clock protein Tim during a key stage of early development. Specifically, EBs formed from ES cells lacking Tim showed reduced caspase activity and failed to cavitate. As a consequence, further development was halted, and the cells present in the failed cavity remained pluripotent. These findings reveal a new function for Tim in the coordination of ES cell differentiation, and raise the intriguing possibility that circadian rhythms and early development may be intimately linked.

## Introduction

Virtually all mammalian cell types express multiple members of the Src protein-tyrosine kinase family, which regulate diverse pathways involved in cell growth, survival, differentiation, adhesion, and migration [1]. The mammalian Src family consists of eight members, some of which exhibit fairly broad tissue distribution patterns in adults (c-Src, c-Yes, and Fyn) with the balance showing more lineage-restricted expression patterns particularly in cells of hematopoietic origin (e.g., Hck and Fgr in myeloid leukocytes, Lck in T-lymphocytes, Blk in B-cells, and Lyn in multiple hematopoietic cell types). Strict control of Src-family kinase (SFK) activity is essential to normal cellular function and development [2], and loss of kinase regulation contributes to several forms of cancer and other diseases [3].

The SFKs exhibit homologous domain organization which includes a myristoylated (and in most cases palmitoylated) N-terminal region, modular SH3 and SH2 domains, a helical SH2-kinase linker, a bilobed kinase (catalytic) domain, and a C-terminal negative regulatory tail. The crystal structures of near full-length versions of c-Src and Hck show that the SH3 and SH2 domains both contribute to negative regulation of kinase activity [4]–[7]. These non-catalytic domains pack against the back of the kinase domain to stabilize a closed, inactive conformation. In this state, the SH3 domain engages the SH2-kinase linker, which adopts a polyproline type II helical conformation typical of SH3 docking sites. The SH2 domain interacts with the C-terminal tail, which is phosphorylated on a conserved tyrosine residue by the independent regulatory kinases, Csk and Chk [8], [9]. Relevant to the current study is the observation that SFK activity must be carefully regulated during mammalian development. While genetic knockouts of individual SFKs produce well-defined phenotypes in most cases, these SFK-null animals remain fertile and viable [10]. In contrast, knockout of Csk, one of the upstream regulators of the Src kinase family, results in embryonic lethality with concomitant upregulation of SFK activity in the non-viable embryos [11].

Recent work has implicated SFKs in mouse and human ES cell growth and differentiation [12]–[14]. In mES cells, seven of the eight mammalian SFKs are expressed and appear to serve non-redundant functions [12]. In this previous study, differentiation of mES cells to EBs was shown to correlate with transcriptional silencing of Hck and Lck, whereas c-Src and Fyn were expressed and remained active in both pluripotent mES cells and differentiated EBs cultured from them [12]. Suppression of all SFK activity with the broad-spectrum pyrrolo-pyrimidine SFK inhibitor A-419259 substantially delayed EB formation from mES cells, and the inhibitor-treated cultures retained the characteristics of pluripotent ES cells [12]. Extending these earlier findings with a chemical genetics approach, our group recently established a non-redundant role for c-Src activity in mES cell differentiation [13]. Here, mES cells were engineered to express an inhibitor-resistant variant of c-Src; when these cells were treated with the inhibitor, they underwent differentiation to primitive ectoderm despite the presence of LIF. Similar experiments with inhibitor-resistant variants of other SFKs had no observable effects. Thus, these studies strongly suggest that c-Src activity alone is sufficient to allow mES cells to exit from the self-renewal program and begin to differentiate. In the present study, we applied a proteomics approach to identify unique binding partners for c-Src in self-renewing vs. differentiated ES cells as a way to define signaling pathways linking Src-family kinases and ES cell fate. These studies identified Tim, a circadian rhythm protein, as a previously unrecognized binding partner and substrate for c-Src and a key regulator of EB formation.

Tim was first described in the context of the *Drosophila* circadian cycle, where clock proteins regulate their own syntheses in a rhythmic fashion [15]. Proteosomal degradation of circadian proteins, including Tim, eliminates negative feedback on transcription and allows RNA levels to rise. Regulation of *Drosophila* Tim levels by targeted degradation is preceded by tyrosine phosphorylation, although the precise kinase(s) involved have not been identified [16], [17]. Although not definitively placed in the circadian pathway in mammals, Tim expression exhibits 24-hour oscillation and it associates with the core clock components Period and Cryptochrome [18], [19]. Furthermore, conditional knockdown of Tim in the rat suprachiasmatic nucleus disrupted the rhythms of neuronal activity and altered levels of clock elements, suggesting a role similar to that of its *Drosophila* counterpart [18]. Mammalian Tim also appears to link the circadian and cell cycles and may have additional functions in DNA damage control [20], [21]. Genetic studies show that Tim is essential for early embryonic development, as homozygous knockout produces early embryonic lethality in mice [22]. In this study, we show that Tim is expressed in mES cells and is essential for the differentiation of ES cells to cystic EBs.

## Results and Discussion

**Figure 1:**
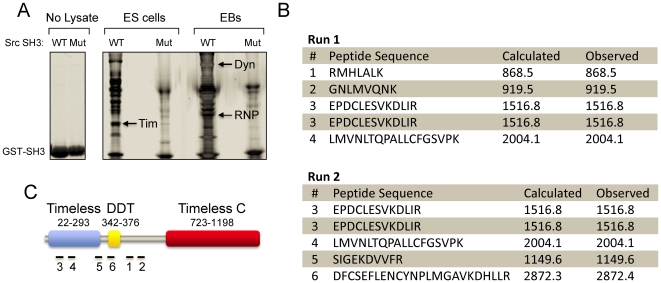
Identification of Src SH3-binding proteins in ES cells and embryoid bodies (EBs). The mouse c-Src SH3 domain as well as a binding-defective control were expressed in bacteria as GST fusion proteins and immobilized on glutathione-agarose beads. Lysates from self-renewing ES cells and 6 day EBs were incubated with the immobilized wild-type and mutant Src SH3 domains, and associated proteins were eluted and resolved by SDS-PAGE (see Materials and Methods). **A**, Image of a Coomassie blue-stained gel of the purified wild-type (WT) and mutant (Mut) Src GST-SH3 proteins (No Lysate). SH3 target proteins captured from ES cell and EB lysates are indicated in the next four lanes. Unique bands were excised and identified via tryptic fingerprinting and MALDI-TOF MS, including the known Src SH3 interacting proteins hnRNP K (RNP) and Dynamin II (Dyn), as well as a fragment of Timeless (Tim; *arrows*). **B**, Details of the MS data obtained for Tim. The excised band from ES cells in Part A was halved and subjected to two separate MS runs. Tim was the top hit from both runs, with two peptides of identical sequence identified in each case. The sequence as well as the calculated and observed masses for each peptide are shown. **C**, The six numbered peptides from Part B map within the N-terminal region of the Tim protein. Three major domains of Tim are illustrated, including the N-terminal Timeless region, the DDT (Domain binding homeobox and Different Transcription factors) domain, and the C-terminal Timeless C region.

**Figure 2:**
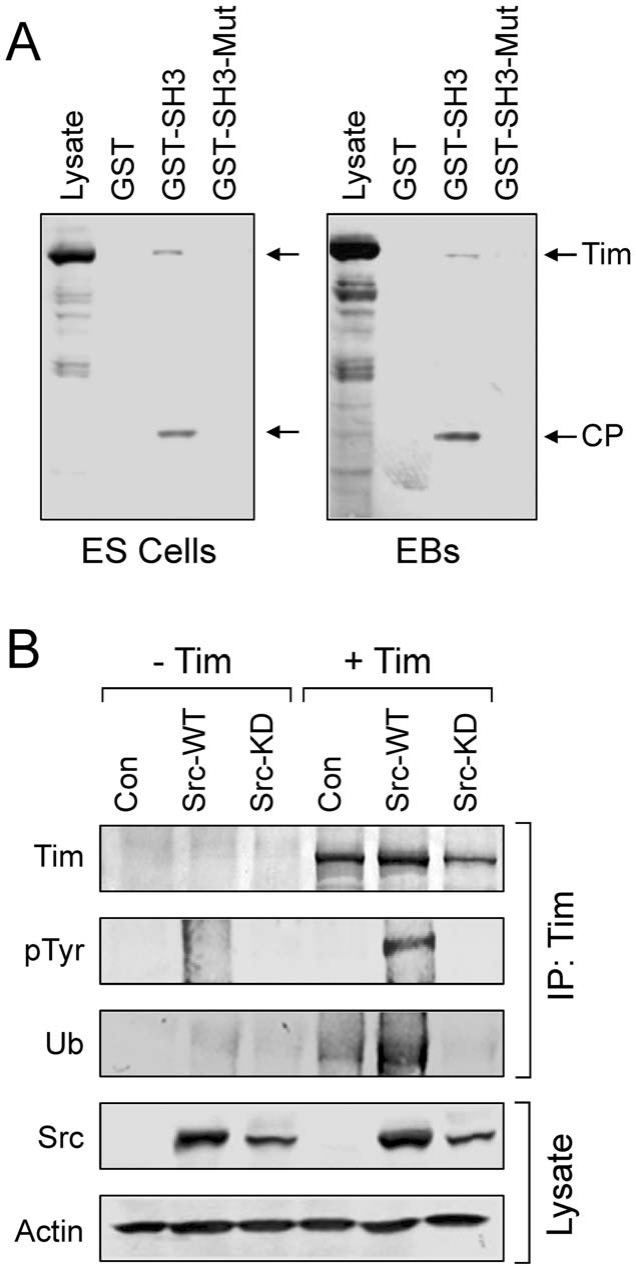
Tim is a c-Src SH3 domain binding protein and substrate. **A**, Lysates were prepared from ES cells and EBs and incubated with immobilized GST, the Src GST-SH3 fusion protein, or the corresponding inactive GST-SH3 mutant. Following washing, bound proteins were separated by SDS-PAGE, transferred to PVDF membranes and probed with an anti-peptide antibody to Tim. Full-length Tim and a discrete cleavage product (CP) were found to associate with the GST-SH3 fusion protein, but not with GST alone or with the mutant GST-SH3 domain. **B**, Tim is a substrate for c-Src. Human 293T cells were transfected wild-type c-Src (Src-WT), a kinase-defective mutant (Src-KD), or with the empty expression plasmid (Con) together with V5 epitope-tagged mouse Tim as indicated. Tim was immunoprecipitated from the transfected cell lysates with a V5 antibody and immunoblotted for Tim protein recovery (Tim), tyrosine phosphorylation (pTyr), and ubiquitin (Ub). Tranfected Src protein expression was confirmed in the cell lysates, with actin as a loading control.

Previous studies summarized above point to the c-Src protein-tyrosine kinase as an important regulator of the earliest stages of mES cell differentiation. These findings led to the question of the signaling pathways controlled by c-Src that account for its role in ES cell fate. To address this question, c-Src target protein capture experiments were performed using an immobilized, recombinant c-Src SH3 domain fusion protein and soluble protein extracts from both self-renewing mES cells as well as differentiated EBs. SH3 domains contribute not only to SFK regulation (see Introduction) but also to substrate recruitment by binding to target proteins containing polyproline type II helices [23]. Unique SH3-interacting proteins were captured by the c-Src SH3 domain but not by an inactive mutant control domain, indicative of specific binding (Fig. 1). Three prominent bands were excised from the gel, digested with trypsin, and identified by MALDI-TOF MS and MS/MS sequencing: 1) Dynamin II, a GTPase involved in vesicular trafficking [24]; 2) hnRNPK, which regulates transcription, pre-mRNA processing, mRNA transport and translation [25]; and 3) a 54 kDa N-terminal fragment of Tim. Both Dynamin II and hnRNPK have been identified previously as c-Src SH3-binding proteins [26], [27], validating our experimental approach. In contrast, the SH3-dependent association of Tim with c-Src or other SFKs has not been reported, suggestive of a novel interaction. To confirm that Tim is an SH3-binding partner for c-Src, SH3 capture experiments were repeated using lysates from ES cells and EBs, followed by immunoblotting with an antibody to the Tim protein. Full-length Tim was captured in each case, as well as a prominent cleavage product that corresponds in size to the fragment originally identified by tryptic fingerprinting (Fig. 2A). In contrast, no binding was detected with the inactive mutant of the c-Src SH3 domain or with GST alone, indicative of a specific SH3-mediated binding event.

We next investigated whether Tim is a substrate for c-Src. For these experiments, full-length Tim was co-expressed with wild-type c-Src or a kinase-defective mutant in the human epithelial cell line, 293T. Tim was then immunoprecipitated from transfected cell lysates, and analyzed for tyrosine phosphorylation by immunoblotting with anti-phosphotyrosine antibodies. As shown in Figure 2B, Tim was strongly phosphorylated in the presence of active c-Src but not with a kinase-dead mutant, demonstrating that Tim is a Src substrate. To investigate a possible relationship between Tim tyrosine phosphorylation and ubiquitylation, as observed previously in *Drosophila* [16], aliquots of the same Tim immunoprecipitates were also immunoblotted with ubiquitin antibodies (Fig. 2B). Co-expression with c-Src strongly enhanced Tim ubiquitylation, suggesting that Src-mediated Tim phosphorylation enhances this process. Interestingly, co-expression of Tim with kinase-inactive c-Src suppressed Tim ubiquitylation below control levels observed in the absence of c-Src expression, suggestive of a dominant negative effect (Fig. 2B).

**Figure 3:**
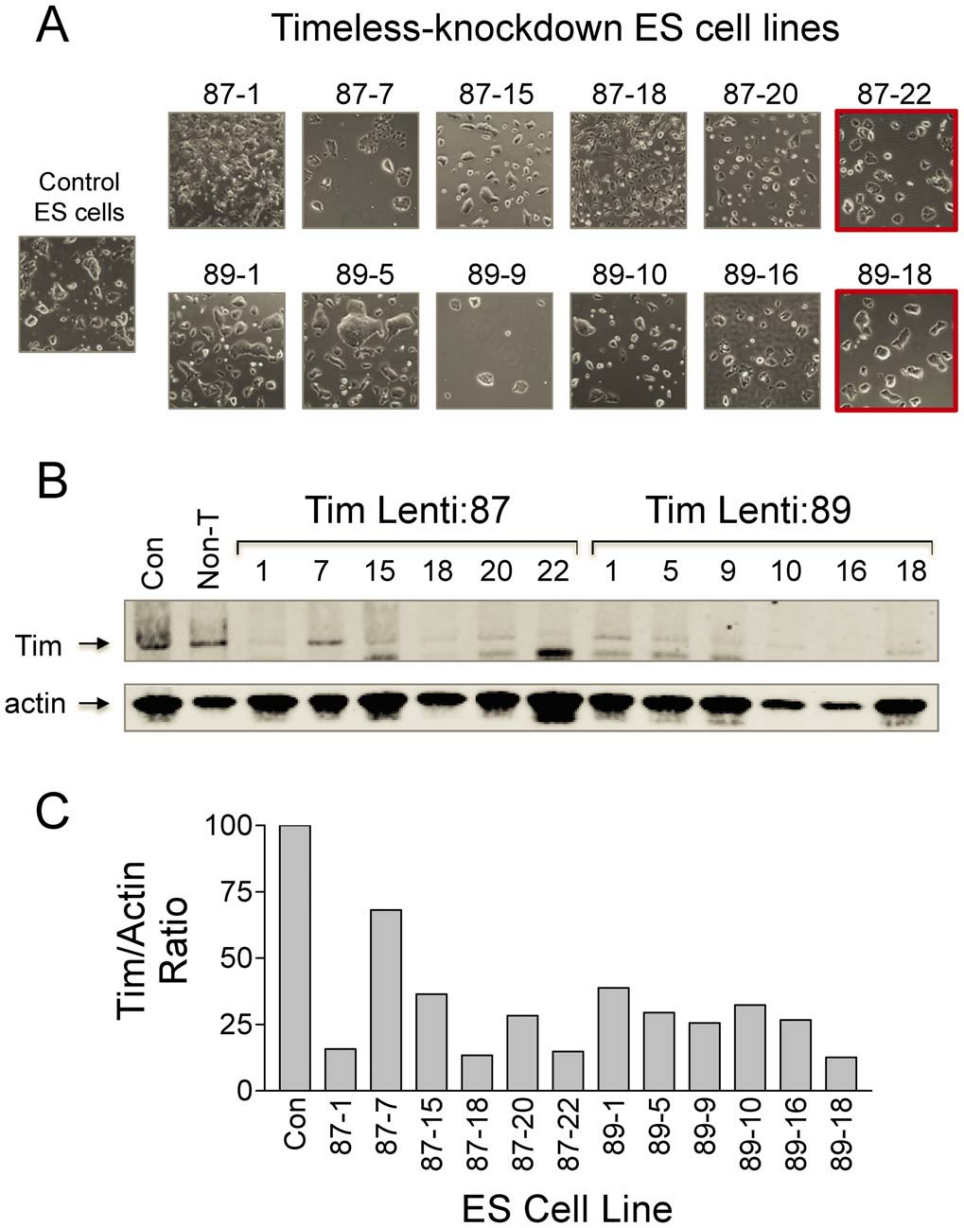
Generation of Tim knockdown ES cell lines. Endogenous Tim expression was suppressed in mES cells by transduction with lentiviral particles carrying shRNA sequences targeting independent regions of the *Tim* locus (Lenti:87 and Lenti:89). Following puromycin selection, 12 undifferentiated colonies were picked, expanded, and the levels of Tim protein expression determined by quantitative immunoblotting. **A**, Morphology of representative Tim-knockdown lines isolated from the Lenti:87 and Lenti:89 ES cell populations. Control ES cell colony morphology is also shown for comparison. **B**, The relative level of full-length Tim in lysates from each of the Tim knockdown lines shown in Part **A** was determined by quantitative immunoblotting (Tim; arrow). Immunoblots were also probed with an actin antibody as a loading control, and the relative levels of each protein were quantitated using the LI-COR Odyssey system and secondary antibodies conjugated to infrared fluorphores. **C**, Bargraph showing the Tim:actin protein ratios. Tim knockdown ES cell lines Lenti:87-22 and Lenti:89-18 were used in subsequent experiments based on unchanged ES cell colony morphology (Part A; red outline) and extent of Timeless knockdown.

To investigate the role of Tim in mES cells, we first over-expressed the protein and observed a reduction in cell viability and enhanced apoptosis compared to untransfected control cells (data not shown). Because of the negative impact of Timeless expression on ES cell viability, we turned to the complementary approach of gene silencing. In these experiments, Tim expression was knocked down by lentiviral transduction of shRNAs targeting two distinct regions of the Tim transcript. Both lentiviral vectors yielded ES cell populations with substantial reductions in endogenous Tim protein levels (data not shown). Six Tim knockdown cell lines were subsequently cloned from each shRNA-transduced mES cell population, and screened for the extent of Tim knockdown by immunoblot analysis (Fig. 3). The two cell lines exhibiting the greatest extent of full-length Tim knockdown without changes in undifferentiated colony morphology were selected for further analysis. These lines were designated as lenti:87-22 and lenti:89-18.

**Figure 4:**
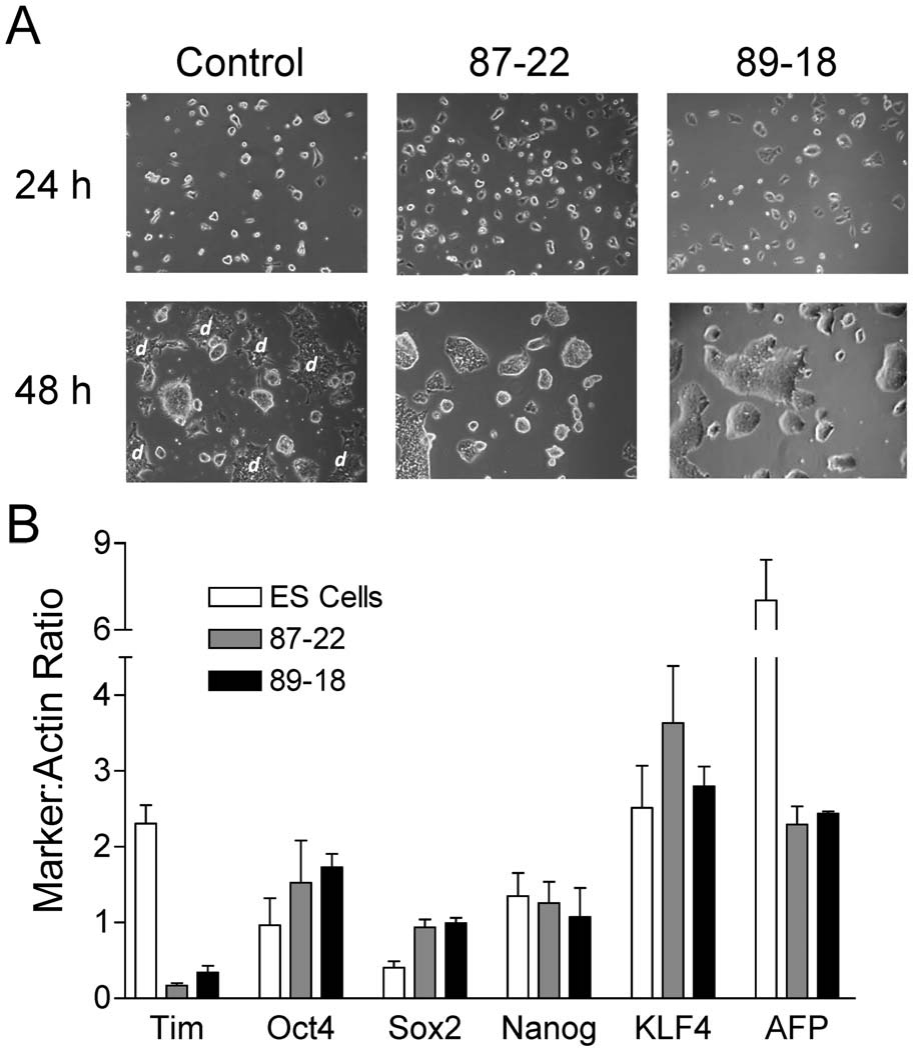
Knockdown of Tim suppresses spontaneous differentiation of mES cells. **A**, Representative images of control and Tim knockdown ES cell lines 87-22 and 89-18 after 24 and 48 h in culture. Note that spontaneously differentiating mES cell clusters are readily apparent in control ES cell cultures by 48 h (flat colonies with ragged edges; “*d*”) but are absent from the Tim knockdown cultures. **B**, Analysis of self-renewal and differentiation marker expression in Tim knockdown cell lines. Expression levels of the self-renewal markers Oct4, Sox2, Nanog, KLF4, the differentiation marker AFP, as well as Tim were assessed by quantitative immunoblotting (LI-COR Odyssey infrared imaging system) of cell lysates from parental ES cells as well as the Tim knockdown ES cell lines 87-22 and 89-18. Actin immunoblots served as loading control. Immunoblots were performed in triplicate and the level of each protein was normalized to actin and is shown in the bargraph as the mean ± S.E.M. Sox2 expression levels were significantly increased in the Tim knockdown cells relative to parental ES cells (p<0.05), while AFP showed a statistically significant decrease (p<0.05). While small increases in Oct4 expression were also observed in the Tim knockdown cell lines, these changes were not statistically significant.

Morphologically, self-renewing cultures of both Tim-knockdown ES cell lines exhibited less spontaneous differentiation when compared to wild-type ES cells (Figs. 3 & 4A). To investigate whether the loss of Tim influenced the expression of self-renewal markers, we examined the levels of Oct4, Sox2, Nanog, and KLF4 by quantitative immunoblotting. As shown in Figure 4B, both Tim-knockdown lines exhibited modest increases in the expression of Oct4 and Sox2, while levels of Nanog and KLF4 were essentially unchanged. Conversely, expression of the differentiation marker AFP was decreased by more than 60% relative to control ES cells. These changes most likely reflect loss of spontaneous differentiation as a consequence of Tim knockdown, although an influence on the ES cell self-renewal program cannot be ruled out.

**Figure 5:**
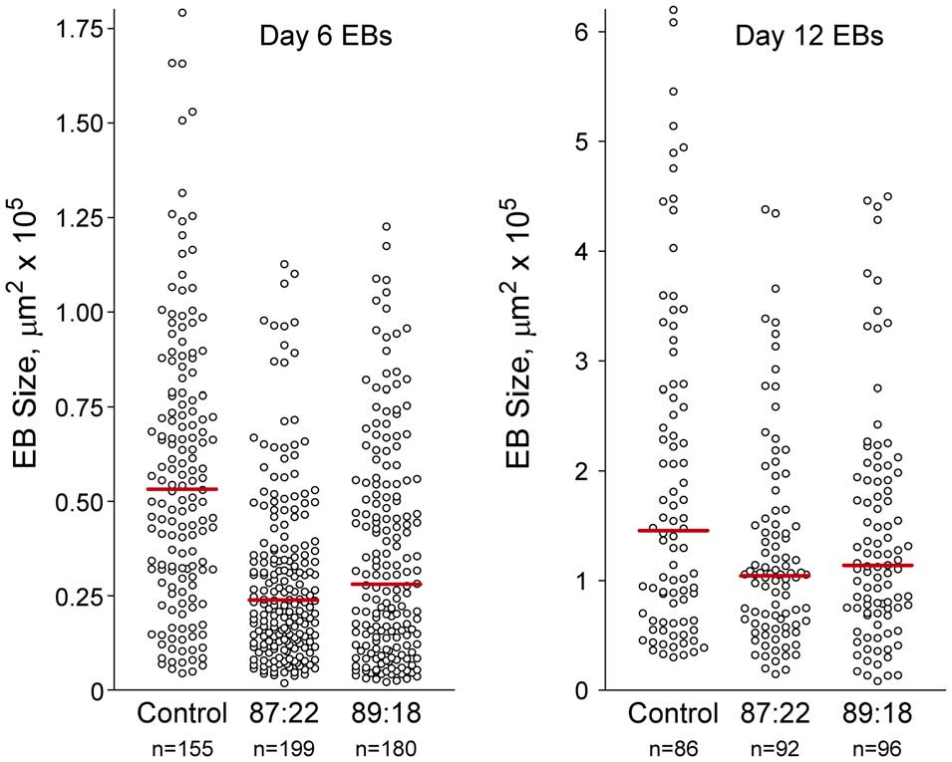
Tim knockdown EBs are smaller and of more uniform size. EBs were grown from control and Tim knockdown ES cell lines Lenti:87-22 and Lenti:89-18 for 6 and 12 days. EBs were fixed, DAPI-stained and imaged with an Olympus F500 confocal microscope. The EB sizes were then estimated by determining the EB surface area from the images using the ImageJ 1.43U software suite. Scattergrams of the resulting data were analyzed using the Kruskal-Wallis test (nonparametric unpaired analysis of variance by ranks; Prizm Software, GraphPad, Inc.) and a significant difference in the median EB size was observed across both groups (P<0.0001). The median EB size is shown in each group by the red bar.

**Figure 6:**
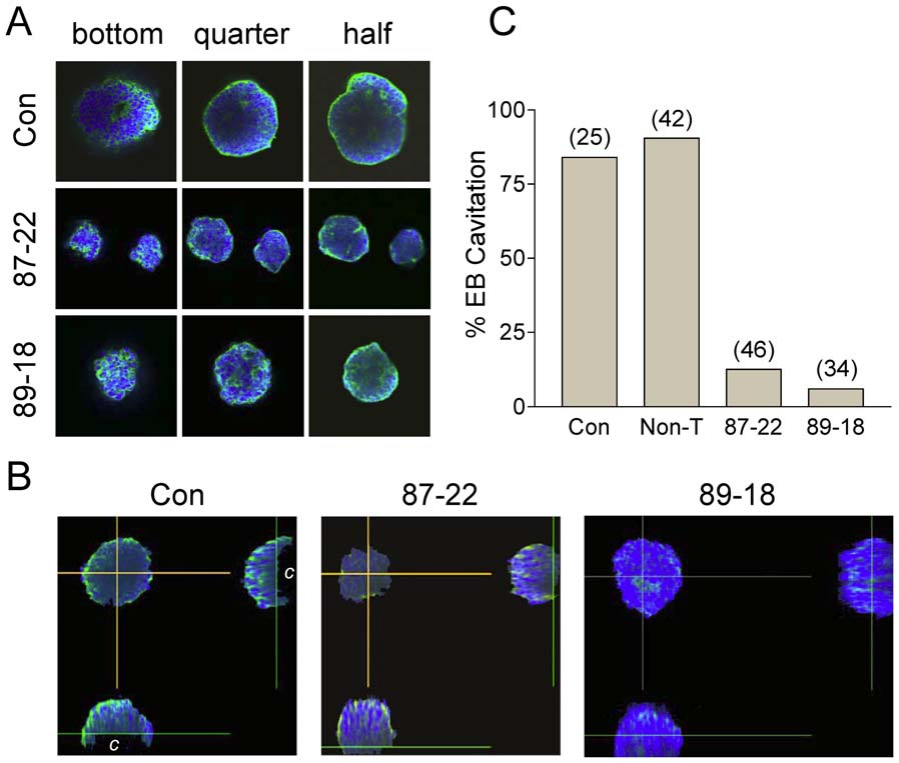
Tim knockdown prevents EB cavitation. EBs were cultured from control and Tim knockdown ES cell lines Lenti:87-22 and Lenti:89-18 for 6 days. Fixed EBs were then stained with DAPI (nuclei; blue) plus Alexa Fluor 488-phalloidin (F-actin; green) and imaged by confocal microscopy using an Olympus Fluoview 1000 confocal microscope. **A**, Optical sections (5 µm) were taken from the bottom through the middle of the EB where cavitation is the greatest. Images of representative sections are shown of the bottom as well as one-quarter and halfway through the EB. **B**, Side profiles from merged images reveal cavitation (“c”) in the control but not the Tim knockdown EBs. **C**, Bargraph shows the percentage of cavitated EBs formed from the parental mES cell line, mES cells transduced with a nontargeted shRNA lentivector (Non-T) as well as the two Tim knockdown ES cell lines.

To determine whether the presence of Tim is essential for early development, we next tested the ability of the Tim knockdown ES cell lines to form EBs. In the EB assay, ES cells are plated in suspension in the absence of LIF, the cytokine required for the maintenance of mES cell pluripotency. Under these conditions, the cells differentiate into organized cysts that recapitulate the initial stages of pre-implantation development, including formation of an endodermal surface layer, differentiation of columnar epithelium, and hollowing out of a central cavity via apoptosis [28], [29]. While wild-type ES cells differentiated into a typical heterogenous EB population with respect to size, both Tim knockdown lines produced smaller EBs of more uniform size (Fig. 5). Under normal conditions, cystic EBs undergo expansion as they differentiate. Thus the consistent formation of smaller EBs from the Tim knockdown ES cells suggested a failure to expand and a possible differentiation defect. To address this issue, we harvested control and Tim knockdown EBs after six days of development and imaged them for cavitation by confocal microscopy. As shown in Figure 6, the number of EBs exhibiting cavity formation was substantially reduced in both Tim knockdown ES cell lines. This result may help to explain the early embryonic lethality previously observed in Tim knockout mice. At ED 7.5, homozygous Tim knockout embryos lack cellular organization, with necrotic cell debris filling the amniotic cavity [22]. As cavitation is a prerequisite for gastrulation, the failure to cavitate resulting from loss of Tim may prevent subsequent development as well.

**Figure 7:**
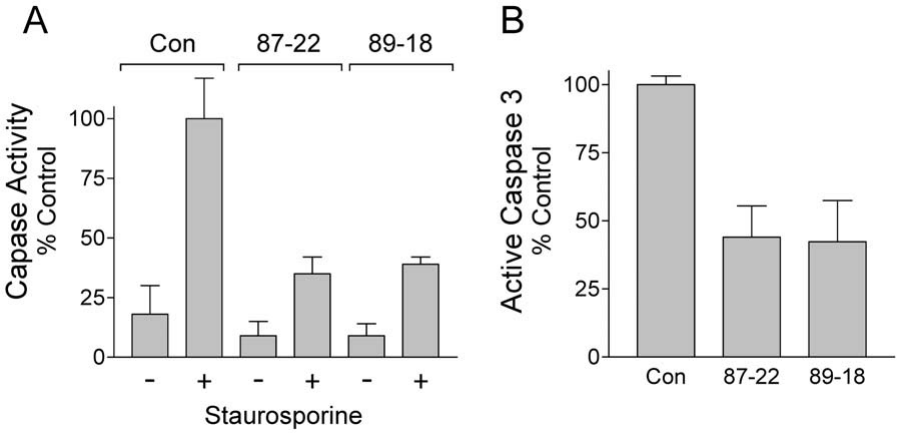
Timeless-knockdown EBs are resistant to apoptosis. **A**, Control and Tim-knockdown EBs were grown for 6 days from the corresponding ES cell lines and incubated in the presence or absence of 0.5 µM staurosporine for 16 h. Cell lysates were analyzed for Caspase 3/7 activity using the Apo-1 assay. The data are normalized to the activity observed with control EBs in the presence of staurosporine. The experiment was repeated three times, and the results are presented in the bargraph as mean percent of control ± S.E.M. **B**, Lysates from staurosporine-treated control and Tim-knockdown EBs were analyzed for the presence of the active form of Capsase-3 by immunoblotting with an antibody specific for the cleaved, active form of this protease. Signal intensities were quantified using the LI-COR Odyssey system, and normalized to control values. The experiment was repeated in triplicate, and the bargraph shows the mean values ± S.E.M. EBs derived from both Tim knockdown cell lines showed a significantly reduced apoptotic response to staurosporine treatment in each of these assays (p≤0.02 in each case).

To determine if loss of cavity formation was linked to an apoptotic defect, Caspase 3/7 activity was determined in wild-type and Tim knockdown EBs following induction with staurosporine (Fig. 7). EBs generated from both of the Tim knockdown mES cell lines exhibited a markedly blunted response to staurosporine in terms of caspase activity as well as production of the cleaved, active form of Caspase 3 (Fig. 7). Interestingly, Caspase activity has been linked to the cleavage of Nanog, one of the core transcription factors known to regulate self-renewal [30]. ES cells lacking the *Casp3* gene are defective for differentiation, which can be rescued by expression of a cleavage-resistant Nanog variant.

**Figure 8:**
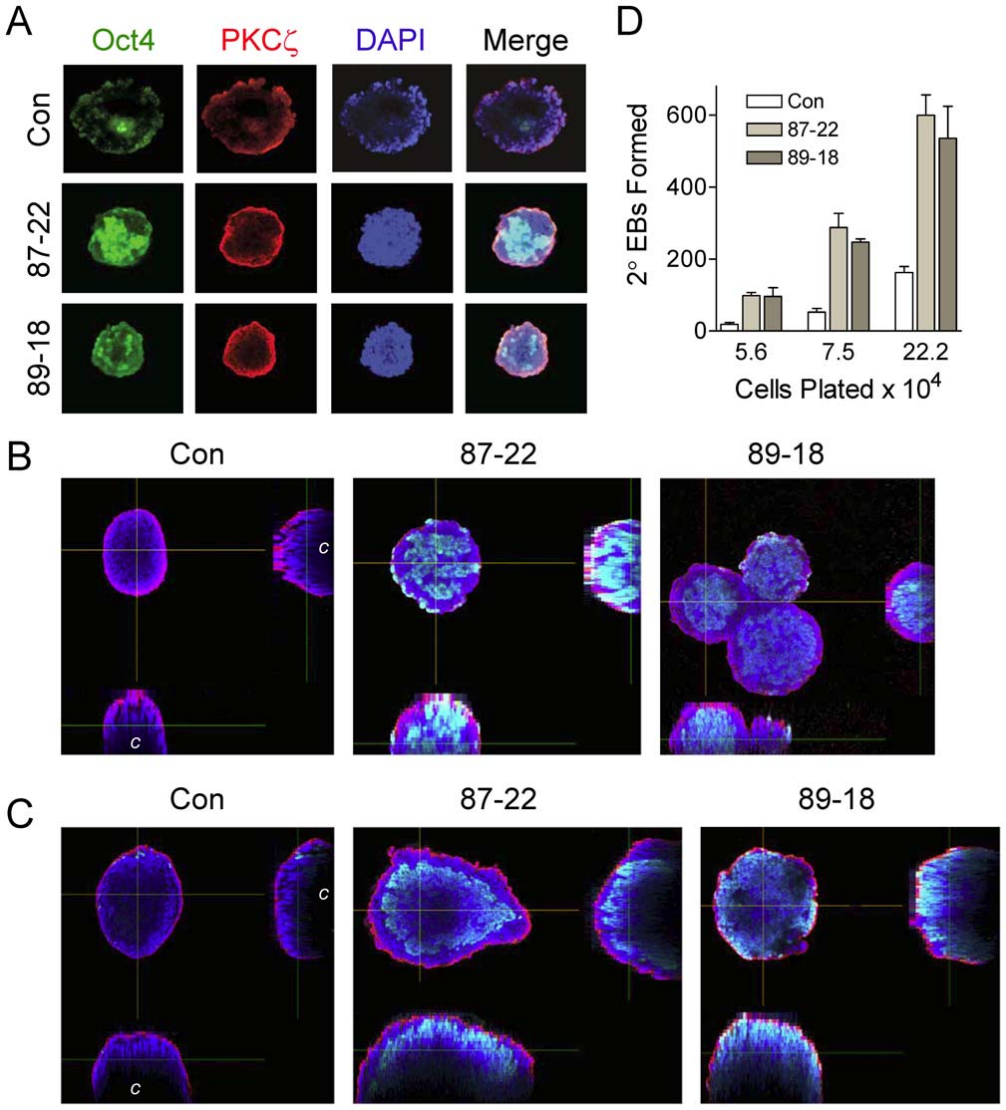
EBs formed from Tim knockdown cells retain pluripotent cells. EBs were cultured from control and Tim knockdown ES cell lines Lenti:87-22 and Lenti:89-18 for 6 and 12 days. **A**, Fixed 6 day EBs were immunostained for the pluripotency marker Oct4 (green) and the zeta isoform of PKC (red) which marks tight junctions in the outer layer of visceral endoderm surrounding the EB. Nuclei were stained with DAPI (blue), and three-color images were obtained by confocal microscopy; a merged image is also shown. Optical sections from the middle of the EB show that cells present in the failed cavity of the Tim-knockdown EBs retain Oct4 staining, indicative of undifferentiated cells. **B**, Side profiles from merged images in part **A**; the location of the cavity in the control EB is indicated with a “c”. **C**, Side profiles from merged images obtained from 12 day EBs and stained as for 6 day EBs. **D**, Secondary EB assay. Six-day EBs from part **A** were trypsinized to single cells, replated in methylcellulose at the cell numbers indicated, and the number of secondary EBs present were counted ten days later. The mean number of secondary EBs formed from each culture ± S.E.M. is shown in the bargraph (n = 8). Both Tim knockdown cell lines produced significantly more secondary EBs than the parental control in each case (p<0.01).

We next investigated whether the cells present in the failed cavity retained characteristics of pluripotent mES cells. Wild-type and Tim knockdown EBs were fixed and immunostained for the pluripotency marker Oct4. In addition, the EBs were immunostained for the zeta isoform of protein kinase C, which is expressed in the tight junctions of the outer visceral endoderm layer and thus defines the outer edge of the EB [31]. Cells remaining in the centers of the Tim knockdown EBs exhibited strong staining for Oct4 after six days, suggesting that the cavity remains filled with undifferentiated cells (Fig. 8A, B). After 12 days, EBs derived from control ES cells showed little Oct4 staining, and 2D projections of the confocal Images revealed substantial cavitation (Fig. 8C). In contrast, 12 day EBs from both Tim knockdown ES cell lines retained very thick walls of Oct4-positive cells, with little to no cavitation evident.

To test whether the Oct4-positive cells present in the Tim knockdown EBs remain pluripotent, we assayed for secondary EB formation. This assay allows for a quantitative comparison of the number of pluripotent ES cells remaining in each culture of primary EBs, as only these cells can give rise to secondary EBs [12], [32]. Six-day EBs from control and Tim knockdown ES cell lines were trypsinized to single cells, plated in methylcellulose and the number of secondary EBs counted 10 days later. EBs from Tim knockdown cells produced substantially more secondary EBs compared to control EBs (Fig. 8D), providing strong evidence that the cells present in the failed cavity remain undifferentiated. These data support a model in which Tim is required for cavitation and removal of pluripotent cells from the developing EB; without Tim, cavitation and subsequent development are arrested.

**Figure 9:**
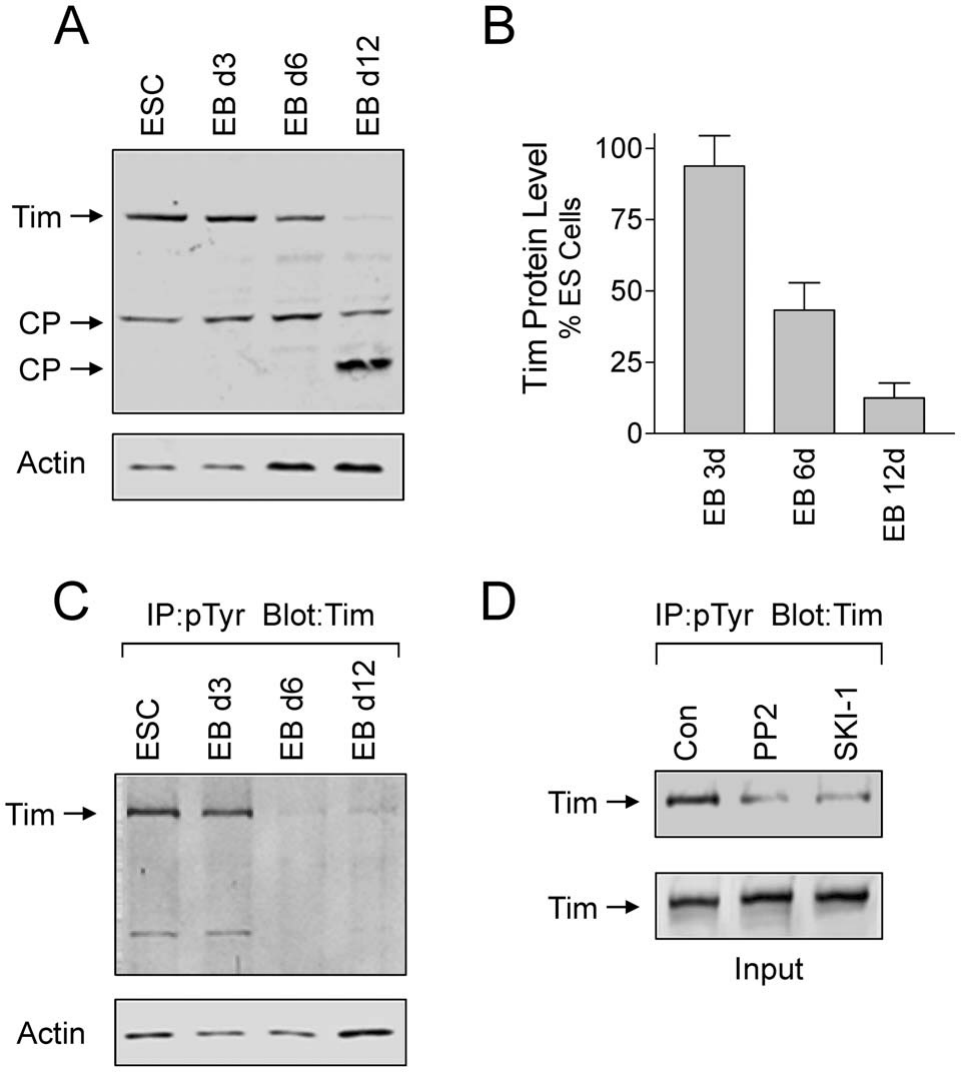
Changes in Tim protein levels and tyrosine phosphorylation during EB formation. **A and B**, Tim protein levels diminish during EB formation. Lysates were prepared from self-renewing ES cells (ESC) and 3, 6, and 12 day EBs, and immunoblotted for Tim and actin protein levels. Full length Tim as well as two possible cleavage products (CP) are indicated by the arrows. Relative band intensities for full-length Tim and actin were determined using ImageJ from four independent experiments and Tim:actin ratios were calculated. The results were normalized to ratios obtained from control ES cells, and are presented in the bargraph as the mean ± S.E.M. The level of Tim was significantly reduced after 6 and 12 days of EB formation (p≤0.01). **C**, Tyrosine phosphorylation of endogenous Tim in ES cells and EBs. Lysates were prepared from self-renewing ES cells and 3, 6, and 12 day EBs, and tyrosine-phosphorylated proteins were immunoprecipitated from protein aliquots and analyzed for Tim by immunoblotting (top panel). Actin blots were performed to verify equivalent levels of input protein for the immunoprecipitation (lower panel). This experiments was repeated three times with comparable results; a representative example is shown. **D**, Inhibition of Tim tyrosine phosphorylation in ES cells by Src-family kinase inhibitors. ES cells were incubated with the Src-family kinase inhibitors PP2 and SKI-1 [12] at 10 µM for 16 h. Tyrosine-phosphorylated proteins were immunoprecipitated and analyzed for the presence of Tim by immunoblotting (top panel). Tim blots (lower panel) verified equivalent levels of Tim in each lysate prior to immunoprecipitation.

In a final series of experiments, we investigated whether Tim protein levels varied as a function of EB formation. Lysates were prepared from self-renewing ES cells as well as 3, 6 and 12 day EBs, and aliquots were analyzed for Tim protein levels by immunoblotting. Overall Tim levels began to decrease after 6 days of EB formation, and were dramatically reduced after 12 days (Fig. 9A and B). The timing of Tim protein loss correlates with cavity formation, which is clearly evident after 6 days and complete after 12 days (Figs. 6 and 8) [28], [29]. We also observed that tyrosine-phosphorylated Tim is present in ES cells as well as 3 day EBs, and diminishes as Tim protein levels decrease (Fig. 9C). Treatment of ES cells with two inhibitors previously shown to block all Src-family kinase activity in ES cells (PP2 and SKI-1) [12] substantially reduced endogenous Tim tyrosine phosphorylation, strongly implicating c-Src or another member of the Src-kinase family as the kinase responsible for Tim phosphorylation (Fig. 9D). This result is consistent with the tyrosine phosphorylation of Tim following co-expression with active c-Src in 293T cells as shown in Figure 2B.

Data presented here provide the first evidence that the circadian rhythm protein Tim also has a central role in one of the first morphogenic changes that accompanies early development. Knockdown of Tim results in ES cells that are capable of forming EB-like structures, but these EBs fail to expand or cavitate and remain filled with undifferentiated cells. While the specific mechanism by which Tim regulates cavitation and subsequent development will require further investigation, our results clearly implicate Tim in the first wave of Caspase 3-dependent apoptosis essential for EB cavitation and morphogenic progression.

In addition to early embryogenesis, other studies suggest that Tim may regulate apoptosis required for organ formation later in development. In the mouse embryonic kidney, for example, strong Tim expression has been observed in regions of active ureteric bud branching. Conditional knockdown of Tim with antisense oligonucleotides in both kidney rudiments and isolated ureteric bud cells profoundly inhibits embryonic kidney growth and ureteric bud morphogenesis [33]. Interestingly, blocking Caspase activity also prevents ureteric bud formation, suggesting that Tim may also regulate apoptotic signals involved in organogenesis [34].

Because Tim was identified as a c-Src SH3 domain-binding protein in ES cells and EBs, Src kinase activity may regulate Tim protein levels and activity during embryogenesis. In support of this hypothesis, we show that full-length Tim is a substrate for c-Src following co-expression in 293T cells, and that Src-mediated phosphorylation correlates with enhanced Tim ubiquitylation. This finding suggests that tyrosine phosphorylation controls Tim protein levels in a manner analogous to circadian control of Tim in *Drosophila* [16]. Furthermore, we observed that endogenous Tim is tyrosine-phosphorylated in self-renewing ES cells and early stage EBs, and that tyrosine phosphorylation precedes a dramatic decrease in Tim protein levels after EB cavitation has been completed. These observations are consistent with the idea that Src-family kinase-mediated phosphorylation regulates Tim ubiquitylation and proteasomal degradation during differentiation of ES cells to EBs. Interestingly, our previous studies have shown that Src-family kinase inhibitors reversibly delay EB formation [12], [13]. Here we show that these same inhibitors block endogenous Tim phosphorylation in ES cells, consistent with a role for a Src-Tim connection in the ES cell to EB transition.

How Tim is linked to the apoptotic machinery and the relationship of that connection to ES cell differentiation is not clear. Clues to a possible mechanism may come from cellular responses to environmental insults, such as DNA damage, which can also trigger apoptosis. Under these conditions, activation of checkpoint pathways initially induces cell cycle arrest, allowing time for DNA repair to occur. However, if DNA damage is severe, apoptosis is triggered instead (reviewed in [35]). In addition to its circadian role, Tim also functions in S-phase checkpoint control and can induce cell cycle arrest by localizing to the stalled replication fork via interaction with Tipin [21]. This observation suggests the possibility that Tim may cause cell cycle arrest in ES cells via a similar mechanism, thus indirectly inducing apoptosis as required for cavity formation.

In summary, our data provide the first evidence that a protein previously implicated in the control of circadian rhythms also has a unique role to play in mES cell differentiation to EBs, two processes that require tight temporal regulation. Remarkably little is known about the presence or role of circadian rhythms in ES cells or in the developing embryo. One very recent study suggests that circadian clock genes are regulated differently in adult versus embryonic cells [36]. These authors showed that although genes associated circadian rhythms are expressed in the developing embryo in vivo, they were not expressed in a synchronized fashion characteristic of circadian cycling. However, when embryonic heart, liver and kidney tissues were placed into culture, rhythmic expression of one of these clock proteins (PER2) was observed. These observations suggest that ex vivo culture of embryonic tissues, as well as in vitro differentiation of ES cells to EBs as described in our study, may require coordination by circadian rhythm proteins. The more general idea that circadian rhythm proteins may coordinate cell lineage and tissue development should be of significant interest to those exploring the directed differentiation of ES cells in vitro. This process often requires EB formation to allow for coordinated differentiation of the three primitive germ layers [29]. Support for this concept comes from recent work by Yagita, *et al*., who showed that circadian feedback loops fail to oscillate in self-renewing cultures of mES cells, but become activated upon differentiation [37]. Interestingly, oscillation was lost again upon genetic reprogramming of the cells back to the pluripotent state, providing a strong connection between circadian oscillations of clock protein promoter activity and differentiation.

## Materials and Methods

### Culture of ES cells and EBs

Culture conditions for ES cells and EB formation have been described previously [12], [13]. Briefly, the D3 line of mouse ES cells was obtained from the American Type Culture Collection (Manassas, VA) and maintained in Dulbecco's modified Eagle's medium supplemented with 15% fetal bovine serum, 2 mM L-glutamine, 1% nonessential amino acids, 1 mM sodium pyruvate, 0.1 mM 2-mercaptoethanol, and 1000 U/ml LIF (Chemicon International). For EB formation, ES cells were plated in growth medium in the absence of LIF in bacterial-grade Petri dishes. To assay for secondary EB formation, primary EBs were trypsinized to single cells and cultured in the presence of 0.3% methylcellulose and secondary EBs counted 10 days later.

### Identification of Src SH3 domain binding proteins in lysates from ES cells and EBs

The SH3 domain of Src was expressed in bacteria and purified as a GST fusion protein as described elsewhere [38], [39]. Briefly, soluble protein extracts were prepared from pluripotent mES cells and 6 day EBs in lysis buffer [10 mM Tris-HCl (pH 7.5), 150 mM NaCl, 5 mM EDTA, 1% Triton X-100] supplemented with 2 mM sodium orthovanadate, 2 mM NaF and Protease Inhibitor Cocktail Set III (Calbiochem). Extracts were clarified by centrifugation and incubated with GST-SH3 fusion proteins immobilized on glutathione agarose beads. Following incubation, the GST-SH3 resins were washed extensively with lysis buffer, and bound proteins were eluted in sample buffer, separated on 4–20% SDS-polyacrylamide gradient gels and visualized with Imperial Coomassie protein stain (ThermoFisher Scientific). To define non-specific binding, capture reactions were performed in parallel with a mutant GST-SH3 fusion protein (W118A) that fails to bind polyproline type II helices typically found in target proteins for SH3 domains. Specific SH3-bound proteins were digested directly from gel slices with trypsin and identified by MALDI-TOF mass spectrometry (Genomics and Proteomcs Core Laboratory, University of Pittsburgh School of Medicine).

### Analysis of Tim phosphorylation and ubiquitylation

A full-length, V5 epitope-tagged mouse Tim cDNA clone [22] was expressed in 293T cells from the mammalian expression vector pCDNA3.1 (Invitrogen) following transient transfection either alone or in the presence of wild-type or kinase-defective c-Src as per our published protocols [12], [13]. Cell lysates were prepared in RIPA Buffer [50 mM Tris-HCl (pH 7.4), 150 mM NaCl, 1 mM EDTA, 1% Triton X-100, 0.1% SDS, 1% sodium deoxycholate] supplemented with 2 mM sodium orthovanadate, 2 mM NaF, and Protease Inhibitor Cocktail Set III (Calbiochem). Tim was immunoprecipitated via the C-terminal V5 epitope tag, and immunoblotted with anti-phosphotyrosine or ubiquitin antibodies. The V5 epitope tag (AB37292) and actin (MAB1501) antibodies were purchased from Chemicon. Ubiquitin (sc-8017), anti-phosphotyrosine (PY99; sc-7020), and c-Src (B12; sc-8056) antibodies were purchased from Santa Cruz Biotechnology.

Endogenous Tim protein levels were determined in lysates of ES cells and EBs by immunoblotting with a polyclonal rabbit anti-peptide antiserum raised against the mouse C-terminal Tim peptide sequence GTPRVHRKKRFQIEDEDD (Tim-P92 antiserum; Bio-Synthesis). To analyze tyrosine-phosphorylated Tim, tyrosine phosphorylated proteins were immunoprecipitated from ES cell and EB lysates using a 1∶1 mixture of the anti-phosphotyrosine antibodies PY20 (BD Biosciences) and PY99 (Santa Cruz). Tyrosine-phosphorylated Tim present in the immunoprecipitates was visualized by immunoblotting with the Tim-92 antiserum. For the inhibitor studies, ES cells were plated on gelatin-coated dishes and allowed to attach for 6 h prior to addition of the Src-family kinase inhibitors PP2 or SKI-1. Each inhibitor was added at 10 µM, a concentration previously shown to block SFK activity in mES cells [12]. Cells were incubated for an additional 16 h followed by analysis of tyrosine-phosphorylated Tim as described above.

### Apoptosis assays

Caspase 3/7 activity was determined in cell lysates using the Apo1 assay (Promega) in response to staurosporine treatment (0.5 µM for 16 h). Levels of active Caspase 3 were determined by immunoblotting cell lysates with an antibody specific for the cleaved, active form of Caspase 3 (Abcam). Band intensities were quantified using the LI-COR Odyssey infrared imaging system as described elsewhere [40].

### Lentiviral shRNA-based knockdown and EB imaging

Lentiviral particles carrying five distinct shRNAs targeting mouse Tim (Mission; Sigma) were used to infect mES cells. Following puromycin selection, the transduced populations were evaluated for Timeless knockdown by immunoblotting. Two of the shRNA lentiviruses (TRCN0000097987 and TRCN0000097989; abbreviated as Lenti:87 and Lenti:89 elsewhere in the text) induced significant reductions in Tim expression in the overall ES cell population. Six undifferentiated ES cell colonies were then cloned from each culture and tested for the extent of Tim knockdown by immunoblotting using the Tim-P92 antiserum. The impact of Tim knockdown on ES cell pluripotency was assessed by immunoblotting of cell lysates with antibodies against the self-renewal markers Oct4 (sc-5279; Santa Cruz), Sox2 (ab59776; Abcam), Nanog (ab70482; Abcam), KLF4 (sc-20691; Santa Cruz) as well as the differentiation marker AFP (sc-8108; Santa Cruz). Immunoblots for actin (mab1501; Millipore) served as loading controls. Additional details of the immunoblotting proctocols have been published elsewhere [12].

ES cells (5×10^5^) were plated on non-adherent plastic petri dishes and grown in suspension in the absence of LIF to promote EB formation as described elsewhere [12], [13]. EBs were fixed 3, 6 and 12 d later in 4% paraformaldehyde, permeabilized in RIP buffer (50 mM Tris-HCl (pH 8), 150 mM NaCl, 1% Nonidet P-40, 0.5% sodium deoxycholate, 0.1% SDS, 1 mM EDTA), and stained with DAPI (Sigma), Alexa Fluor 488-phalloidin (Molecular Probes) as well as antibodies for Oct4 (sc-5279; Santa Cruz) and the zeta isoform of PKC (sc-216; Santa Cruz) based on the methods of Denham et al. [41]. Optical sections were captured using an Olympus Fluoview 1000 confocal microscope; additional details of the imaging techniques are provided in the Figure legends.

### Statistical analyses

Statistical significance was determined using Student's t-tests where appropriate. Differences in EB size were assessed using the Kruskal-Wallis nonparametric unpaired analysis of variance by ranks. Statistical determinations were performed using the Prism Software package (GraphPad, Inc.).
